# Effect of diode laser biostimulation on implant stability, post-surgical inflammation, and bone healing in immediate implant in the maxillary premolars. A randomized double-blind trial

**DOI:** 10.1007/s00784-026-06847-2

**Published:** 2026-04-14

**Authors:** Basma Ashraf Kassem, Saeeda Mahmoud Osman, Yehia El-Mahallawy

**Affiliations:** Oral and Maxillofacial Surgery Department, Faculty of Dentistry, Alexandria University, Champlion st, Azrite, Alexandria, Egypt

**Keywords:** immediate implant placement, photobiomodulation therapy, laser, implant stability

## Abstract

**Introduction:**

The stimulatory effects of laser Biostimulation on immediate implant clinical wound healing and radiographic bone quality analysis is with insufficient evidence.

**Aim:**

To evaluate the effect of low-level diode laser as a biostimulatory bolster for immediate implants in maxillary premolars.

**Materials and methods:**

The study was conducted as a double-blinded randomized clinical trial on 20 patients with unrestorable maxillary premolars with a Type I socket. Patients were divided into two groups, where those who received immediate implant with laser biostimulation were assigned to the study group, while a sham laser application was used in the control group. Implant stability was appraised using radiofrequency analysis. Radiographic evaluation was conducted using an immediate post-operative and after 4-months tomographic scans.

**Results:**

Implant stability analysis showed a significant difference in the reported secondary stability values in the irradiated group. The healing index quantitative assessment of wound healing, reported significantly higher scores compared to the control group at all follow-up points. Radiographic bone density and labial plate thickness showed statistically insignificant differences.

**Conclusion:**

The overall success rate, secondary implant stability, and radiographic performance were comparable to those of the control group. These findings suggest that the biostimulatory effects of the laser are limited in the long term, offering no significant additional benefits over conventional methods.

## Introduction

Alveolar ridge resorption following tooth loss is one of the detrimental consequences of the healing process, where ridge width can be reduced by up to 50% in the first year after tooth loss. Two-thirds of this reduction occurred within the first three months [1]. Immediate implant rehabilitation is gaining popularity as the quintessential treatment modality owing to its ability to overcome the unfavorable alveolar bone dimensional changes, which allows lesser buccolingual bone reduction and wider crest when compared to the delayed implant placement protocols [2]. Furthermore, immediate implant rehabilitation with the utilization of direct customized provisionalization has the added advantage of a substantial reduction in overall procedural rehabilitation time and the psychological impact on some patients [3, 4].

The predictability of the immediate placement procedure depends on numerous soft and hard tissue circumstances for the achievement of the desired outcome. Intact socket walls, sufficient apical bone stock, thick gingival biotype, and absence of infection are some of the factors to be considered during preoperative socket classification and case selection. The literature contains a plethora of adjunctive modalities and trials that were implemented for increasing the predictability of immediate implant rehabilitation [5, 6].

Laser irradiation is introduced as a non-invasive technique that uses low-power radiant energy to improve wound healing. Histomorphometry investigation verified the power of Low-Level-Laser Therapy (LLLT) to improve wound healing, collagen synthesis, and fibroblast multiplication. Moreover, LLLT improves local blood circulation, accelerates activation processes, reduces the risk of infection, improves metabolic activity, and accelerates healing of damaged tissue [6–8].

The laser biostimulatory effect on bone healing is known for its osteogenesis-inductive effect, and it has been successfully used in the acceleration of orthodontic tooth movement and the improvement of post-extraction and bone fracture healing. Karakaya et al. demonstrate that the stimulatory impact of low-laser irradiation on bone-wound healing is linked to fibroblasts and osteoblasts proliferation during mesenchymal development, which improves bone collagen fiber density, stimulates bone matrix production, and expedites bone healing [9]. Furthermore, the increased tissue vascularization releases mediators, which enhance the osseointegration of implanted hardware.

This study aimed to evaluate the effect of low-level diode laser bio-stimulation as an adjunctive biostimulation for immediately placed implants in the maxillary premolar-zone. The primary objective of the study was the assessment of the biostimulation effect on the implant stability of immediately placed implants in the maxillary premolar-area. The secondary outcome of the study was to evaluate the clinical and radiographic performance of maxillary premolars’ immediate implant placement with low laser biostimulation and compare it to the conventional technique.

## Materials and methods

### Study design

**Fig. 1:**
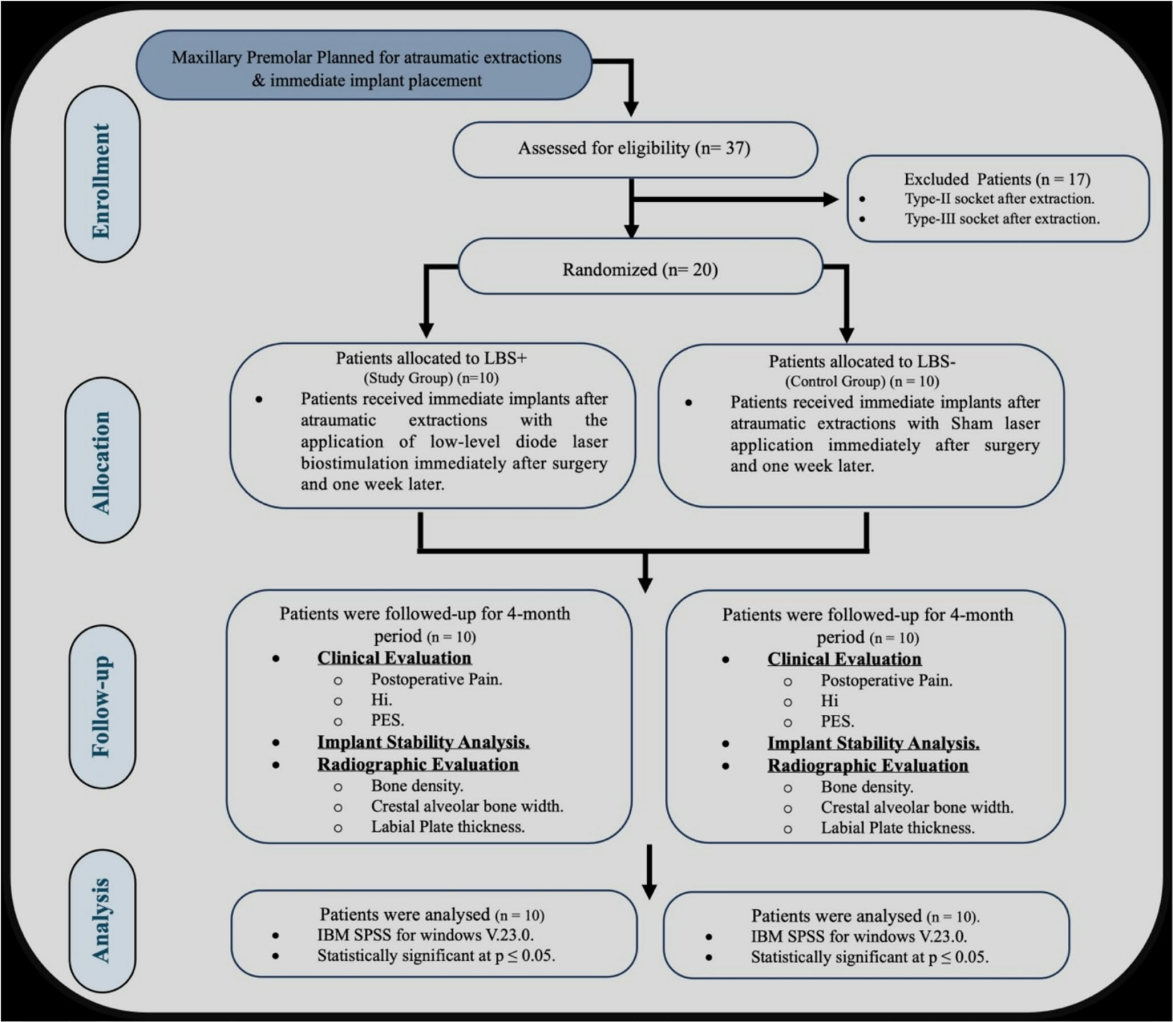
CONSORT Flowchart

The study was conducted as a randomized, double-blinded, controlled clinical trial. The CONSORT guidelines were abided during the conduct of this trial for the evaluation of the biostimulation effect of low-level laser therapy (LLLT) as an adjunct to immediate implant placement in the maxillary premolars. (http://www.consort-statement.org) (Fig. 1). A sample size of 20 patients (10 / group) was computed using Rosner’s method [10] and calculated by G*Power 3.1.9.7 [11]. The sample size was based on Fahim et al. report of the difference in independent means with an effect size of 1.483, and taking dropouts into consideration [12].

### Patients’ selection

A total of 20 patients with unrestorable, badly decayed upper premolar teeth were selected for this study from those who were referred to the Department of Oral and Maxillofacial Surgery, Faculty of Dentistry, Alexandria University. Selected Patients were with sufficient bone quantity to receive the dental implant and a thick gingival biotype based on the gingival biotype [13], Elian’s Type-I [14], and Chang’s Type-I socket [15] classifications. Those with adequate oral hygiene or willing to improve their oral hygiene to ensure uncompromised wound healing were included in the study. Patients with uncontrolled medical conditions that can affect surgical outcome or bone and wound healing were excluded from the study, along with those with periapical pathology that may contraindicate immediate implant placement. Patients with parafunctional habits were also excluded from the study. All selected patients were informed about the details of the study and signed an informed consent. Approval of the Research Ethical Committee was obtained before starting the study, which was conducted under the doctrines of the Helsinki guidelines (IRB:0811 − 012/2023/IORG:0008839) (ClinicalTrials.gov/NCT07044245/2025-6-27).

Selected patients in this study were randomized and allocated into 2 equal groups. The Study Group included those who received immediate implants after atraumatic extractions with the application of LLLT biostimulation (*LBS+ Group*). The Control Group included participants who received immediate implants after atraumatic extractions without laser application (*LBS- Group*). The study was fashioned in a double-blinded manner, as a sham laser application was used for the patients in the control group, and the outcome assessor was blinded. Patient allocation was performed in a 1:1 ratio, using sequentially numbered, opaque, sealed envelopes (SNOSE) for concealment. Computer-based randomization was conducted in the study (http://www.randomizer.org/). Laser application in the study group was performed using a Semiconductor diode laser with a wavelength of 660-nm, and an 8-mm MultiTip (Therapy Light Guide) (SiroLaser blue, Dentsply, Sirona, Charlotte, NC, USA). All the patients in both groups received a similar implant design for standardization (Nuvo- JJGC Indústria e Comércio de Materiais Dentários S.A, Brazil).

### Preoperative assessment

Patients’ medical history was collected preoperatively to exclude patients with medical problems or bad habits that impair bone integration. Extraoral examination, inspection, and palpation for the presence of any swelling or signs of infection, and intraoral examination, to detect bone integrity, any abnormality in bone contour, and assess the ridge condition, were performed. A Cone Beam Computed Tomography (CBCT) scan was obtained for the patients before the surgery to evaluate bone height and width and determine the size of the implant to be placed, and for socket classification. Preoperative scaling and root planning were performed, and oral hygiene recommendations and instructions were given to all patients.

### Surgical phase

The operating field was scrubbed using povidone-iodine scrub solution, and all patients were treated under local anesthesia using 4% Articaine + 1:100,000 epinephrine (Alexadricaine: Alexandria, Egypt). A traumatic extraction of badly-destructed maxillary premolars was performed. The integrity of the socket was further clinically gauged and checked to see if it fit the inclusion criteria, as those with bony deficiencies were excluded from the study. The clinically confirmed Type-I sockets were included in the study, where their sockets were irrigated with normal saline to remove any debris before drilling for the implant. Sequential drilling for the implant is done as recommended by the manufacturer. This was followed by Implant placement in a 2-mm sub-crestal position. The smart-peg was screwed to the implant fixture, then the Osstell device was used, holding its probe close to the smart peg without touching it. The device beeps, displaying the implant stability quotient (ISQ) on its screen for the assessment of the implant’s primary stability. Customized healing abutments were fabricated, polished, and kept out of occlusion for all of the patients in this study. Determination of group allocation was conducted using the on-site computer-based software and the SNOSE concealment method.

For patients in the *LBS+ group*, low-level biostimulation was performed in two consecutive sessions, each of 100s. The first session was carried out immediately after surgery time, after implant placement, using a diode laser Photo-biomodulation tip in a continuous wave and non-contact mode. Laser application parameters were set at 660-nm wavelength, 100 W power, 6 J energy, and 12 J/ cm^2^ fluence, using the MultiTip, with 8-mm tip diameter and 0.5-cm^2^ tip area. The laser was applied at two points: the buccal side and the palatal side. The laser was set at the infrared light mode, which was positioned to allow light to enter perpendicularly to the longitudinal axis of the dental implant at 5-mm from the bone crest. The second session was performed on the 7th postoperative day of the surgery, using similar application parameters [16, 17]. In each session, safety measures were taken with both the patients and surgeons wearing dark protective glasses [18].

For patients in the control group, a sham laser application was performed in two sessions to ensure proper participant blinding. For patients in both groups, the surgical procedure was performed by a single operator (B.K.), while another assessor was assigned for the assessment of the study outcomes and to ensure blinding (Y.E.)

### Implant stability assessment and prosthetic phase

Secondary implant stability analysis was performed 4-months after implant placement. Radio frequency analysis (RFA) was performed using the Osstell device (Integration Diagnostic Ltd. Company, Sävedalen, Sweden). Primary and secondary implant stability were analyzed and compared. The use of the Osstell and ISQ values was obtained by a blinded operator (Y.E.). The final prosthesis was delivered 4-months after implant placement for all of the enrolled patients [19].

### Clinical wound healing assessment

Clinical follow-up assessment was performed at 7, 14, and 21 days post laser irradiation. Postoperative pain was evaluated through a 10-point Visual Analogue Scale (VAS) [20]. A subjective evaluation of the presence/absence of postoperative complications was performed through checking the implant site for pain, swelling, discomfort, redness, warmth, pus discharge, and any other infection-related signs and symptoms, and wound dehiscence [17]. This was performed to assess the early post-operative inflammation through a subjective and dichotomous visual examination (Yes/No). An objective assessment tool was used for the assessment of wound healing using the Landry healing index (Hi), which grades the wound on a scale of 1–5, where 1 indicates very poor healing, and five indicates excellent healing [17, 21]. A final assessment was done after 4-months at the time of final restoration insertion using the Pink Esthetic Score (PES) for the assessment of implant crown as well as soft tissue esthetics [22].

### Radiographic assessment

A CBCT scan was obtained immediately after surgery and four months post-operatively to assess bone density. The bone density was measured within 3 predetermined fixed points around the implant (buccal, lingual, and apical), and the immediate postoperative bone density measurements were taken as a reference point [23]. The width of the alveolar process at the crestal edge of the bone was measured in the preoperative and the 4-month scans to determine the buccopalatal ridge dimensions using the measurements tool on the “On-Demand 3D App.” Software [24].

Assessment of the thickness of the labial bone was performed in the preoperative and the 4-month scans to evaluate the overall survival of the labial plate following the atraumatic extraction and immediate implant placement [1]. Thickness was obtained from the cross-sectional CBCT cut in the preoperative recording at three levels: crestal labial thickness, middle labial thickness, and apical labial thickness. Measurements were obtained from the outer root surface, in the preoperative record, and from the outer surface of the implant, in the 4-month scan, to the outer buccal bone surface [1]. Differences between the two scans were calculated and analyzed.

### Statistical analysis

The IBM-SPSS V.23 was used for the statistical analysis of the data and data normality analysis using the Shapiro-Wilk test and Q-Q plots (Armonk, NY: IBM Corp, released 2011). The Mann-Whitney U test was used for intergroup comparisons, while the Wilcoxon Signed Rank test and Friedman test, followed by pairwise comparisons with Bonferroni correction, were employed for intragroup comparisons. Qualitative variables were analyzed using Pearson’s Chi-square. Percent change was calculated using the following formula: ((Values after 4 months – Values at baseline) / Values at baseline) x 100. All tests were two-tailed, and the significance level was set at p value < 0.05.

## Results

A total of twenty patients who presented with a maxillary premolar tooth indicated for extraction and immediate implant replacement participated in the current study, with a 0.428:1 male-to-female ratio and a mean age of 38.00 ± 5.82 years. Patients were divided into two equal groups: a group with laser biostimulation (LBS+) and a control group with sham laser application (LBS-).

### Implant stability analysis

Resonance Frequency Analysis and its equivalent Implant Stability Quotients (ISQ) were measured for the assessment of the primary implant stability, intraoperative, and the secondary implant stability, 4-months from implant placement at the time of final restoration insertion. Intergroup comparison showed a statistically significant difference in the reported secondary stability values in the LBS+ group (Table 1). The percentage of change of the achieved secondary stability from the primary stability reported was 2.3-times higher in the LBS+ group (*P* < 0.00^*^).

**Table 1:** Implant stability analysis (ISQ) in the laser-stimulated and the control groups

ISQ		LBS+ (*n* = 10)	LBS- (*n* = 10)	*p* ^1^
Baseline Primary Implant Stability	Mean ± SD.	68.40 ± 1.08	66.60 ± 3.57	0.144
	Median.	68.00	68.00	
	Min – Max.	67.00–70.00	60.00–69.00	
4-month Secondary Implant Stability	Mean ± SD.	78.20 ± 3.49	70.60 ± 2.17	< 0.001*
	Median.	79.00	71.00	
	Min – Max.	72.00–82.00	67.00–73.00	
*P* ^2^		< 0.001*	< 0.001*	
Percentage of change (%)	Mean ± SD.	14.30 ± 4.12	6.14 ± 3.13	< 0.001*
	Median.	16.18	5.80	
	Min – Max.	7.46–18.84	2.90–11.67	

ISQ: Implant Stability Quotent; LBS+: Study group with laser application; LBS-: Control group with sham laser application; *P*^1^: Mann-Whitney U test; *P*^2^: Wilcoxon Signed Rank test; *Statistically significant difference at *P*-value < 0.05

### Clinical evaluation

**Fig. 2:**
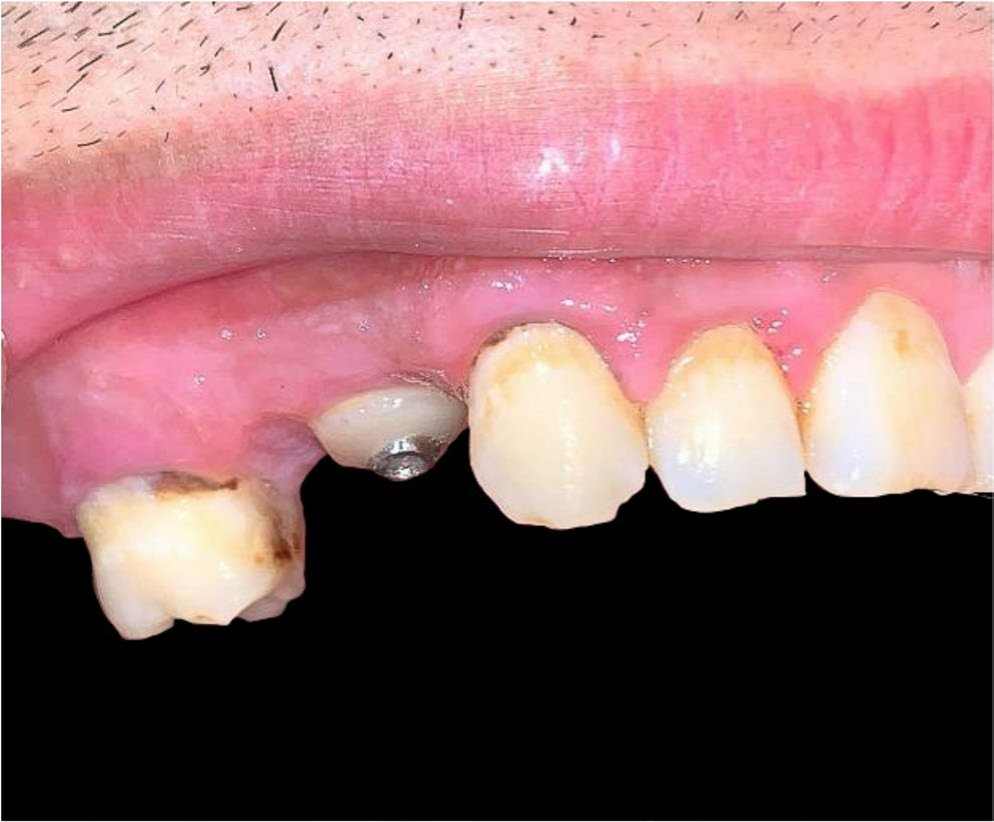
Clinical assessment of cases in the LBS+ group using the Hi, showing excellent healing (score 5) with the utilization of customized healing abutment

At the end of the clinical follow-up period, all patients in both groups experienced a statistically significant improvement in the reported VAS-score (*P* < 0.001^*^). Incidence of postoperative complications was evaluated in 7,14 & 21 days after surgery. In the early evaluation session, none of the patients reported any signs of inflammation or wound healing complications in the LBS+ group. A significant difference is reported in the 7-day evaluation setting, as the control group reported 4 implants with signs of inflammation (*P =* 0.011^*^). However, by the 14 & 21-day sessions, no patients in either group showed signs of wound healing complications. The Landry Healing Index (Hi) quantitative assessment of wound healing reported significantly higher scores compared to the control group at all follow-up points, indicating a substantial improvement in early wound healing (Table 2) (Fig. 2).

**Table 2:** Landry Healing Index quantitative analysis (Hi) in the laser-stimulated and the control groups.

Hi		LBS+ (n=10)	LBS- (n=10)	P^1^
7-Days	Mean ±SD	4.60 ±0.52	2.40 ±0.52	<0.001*
	Median	5.00	2.00	
	Min – Max	4.00 – 5.00	2.00 – 3.00	
14-Days	Mean ±SD	4.80 ±0.42	3.00 ±0.00	<0.001*
	Median	5.00	3.00	
	Min – Max	4.00 – 5.00	3.00 – 3.00	
21-Days	Mean ±SD	5.00 ±0.00	4.60 ±0.52	0.029*
	Median	5.00	5.00	
	Min – Max	5.00 – 5.00	4.00 – 5.00	
P^2^		0.051	<0.001*	

Hi: Landry Healing Index; LBS+: Study group with laser application; LBS-: Control group with sham laser application ; P^1^: Mann-Whitney U test; P^2^: Wilcoxon Signed Rank test; *Statistically significant difference at P-value <0.05.

Aesthetic evaluation of the implant-supported single crowns was conducted using Pink Esthetics Score (PES), which reported an insignificant intragroup difference (*P* = 0.737). The mean reported PES value was 10.60 ± 0.84 and 10.60 ± 0.52 in the LBS + and the LBS- groups, respectively.

### Radiographic evaluation

**Fig. 3:**
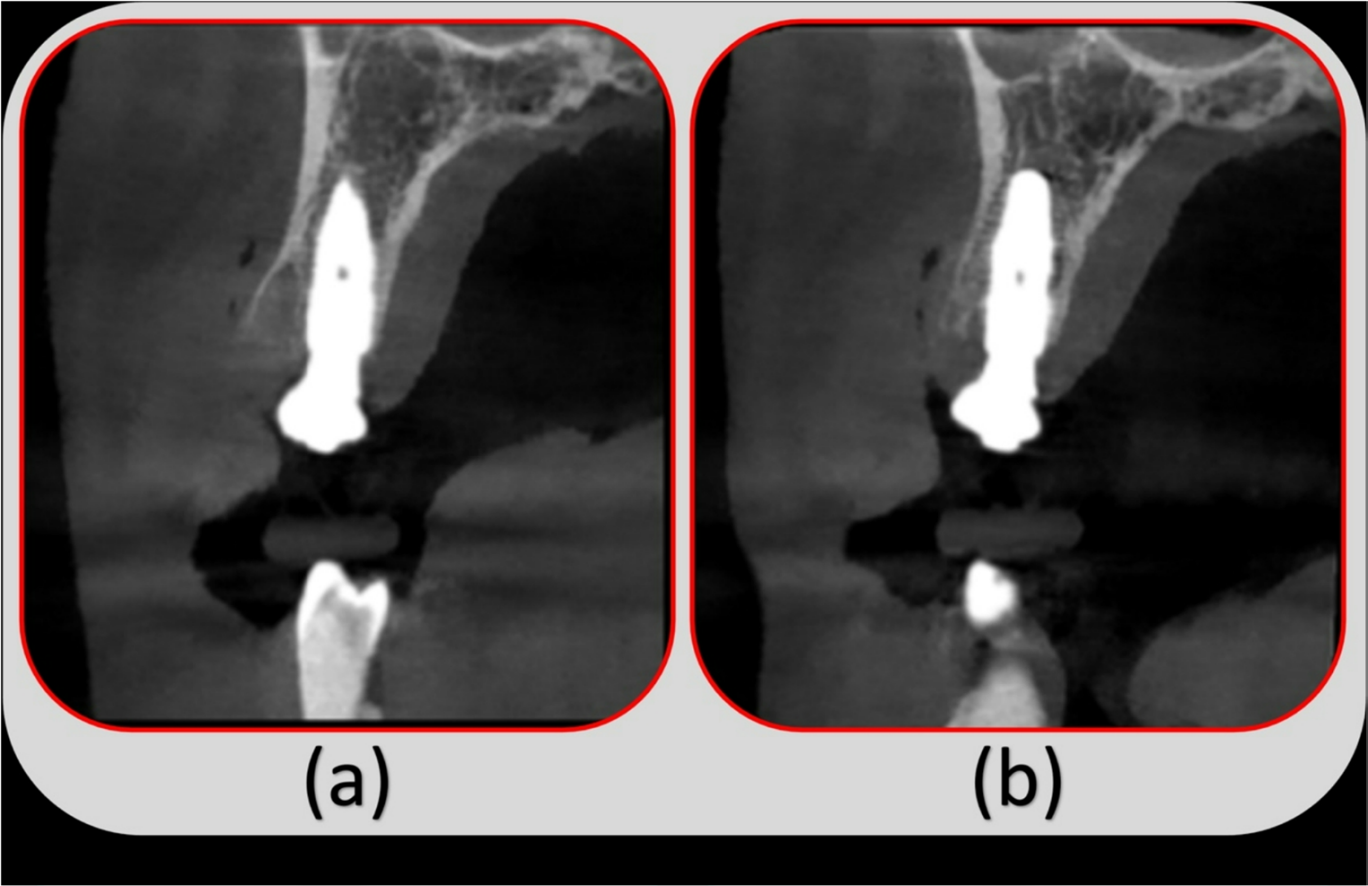
Radiographic appraisal for the case in the LBS+ group. (**a**), Cross-section view of the immediate postoperative CBCT. (**b**) Cross-section view of the 4-month postoperative CBCT

Bone density was evaluated in the immediate postoperative scan and compared to the values reported in the 4-month postoperative scan. Inter-group comparison reported an insignificant difference in the calculated mean immediate postoperative and the 4-month postoperative bone density values (*P* = 0.181) (*P* = 0.058). Both groups exhibited significant intra-group bone density improvements in the 4-month values (*P* < 0.001^*^ for both), with a comparable increase percentage (*P* = 0.710) (Table 3). The Crestal bone width of the Alveolar Process (CBW) was measured in the preoperative and the 4-months scans (Fig. 3). The LBS+ group showed a significantly lower CBW-reduction percentage (3.88% & 7.27% in the LBS + and the LBS- groups, respectively) (*P* < 0.001^*^) (Table 4).

**Table 3:** Bone Density (GSV) analysis in the laser-stimulated and the control groups

Bone Density (GSV)		LBS+ (*n* = 10)	LBS- (*n* = 10)	*p* ^1^
Baseline Immediate Postoperative Scan	Mean ± SD.	651.20 ± 89.38	608.20 ± 39.38	0.181
	Median.	656.00	622.00	
	Min – Max.	556.00–796.00	547.00–651.00	
4-month Postoperative Scan	Mean ± SD.	776.80 ± 85.37	719.40 ± 27.20	0.058
	Median.	789.00	728.00	
	Min – Max.	685.00–886.00	689.00–756.00	
*P* ^2^		< 0.001*	< 0.001*	
Percentage of change (%)	Mean ± SD	19.71 ± 5.00	18.65 ± 7.33	0.710
	Median	20.27	19.06	
	Min – Max	11.31–13.73	6.14–25.96	

GSV: Grey Scale Value; LBS+: Study group with laser application; LBS-: Control group with sham laser application; *P*^1^: Mann-Whitney U test; *P*^2^: Wilcoxon Signed Rank test; *Statistically significant difference at *P*-value < 0.05

**Table 4:** Crestal bone width analysis (mm) in the laser-stimulated and the control groups

CBW (mm)		LBS+ (*n* = 10)	LBS- (*n* = 10)	*p* ^1^
Baseline Immediate Postoperative Scan	Mean ± SD.	7.58 ± 0.45	7.59 ± 0.69	0.976
	Median.	7.53	7.52	
	Min – Max.	6.94–8.25	6.60–8.67	
4-month Postoperative Scan	Mean ± SD.	7.29 ± 0.53	7.04 ± 0.69	0.377
	Median.	7.20	7.02	
	Min – Max.	6.54–8.01	6.03–8.10	
*P* ^2^		< 0.001*	< 0.001*	
CBW-Reduction %	Mean ± SD.	3.88 ± 1.96	7.27 ± 0.87	< 0.001*
	Median.	2.91	6.66	
	Min – Max.	1.54–6.37	6.57–8.64	

CBW: Crestal Bone Width; mm: millimeter; LBS+: Study group with laser application; LBS-: Control group with sham laser application; *P*^1^: Mann-Whitney U test; *P*^2^: Wilcoxon Signed Rank test; *Statistically significant difference at *P*-value < 0.05

**Fig. 4:**
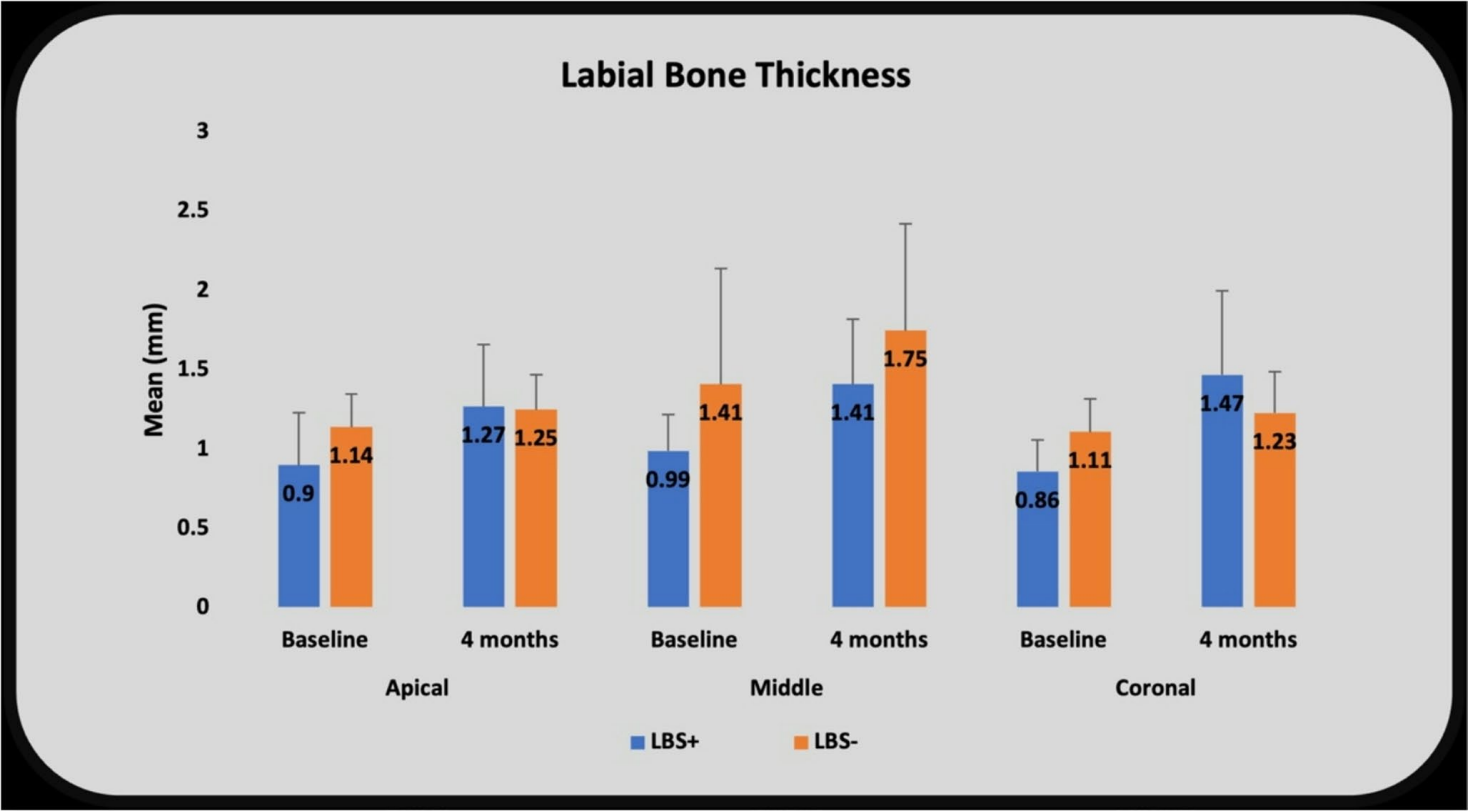
Labial Bone Thickness analysis (mm) in the laser-stimulated and the control groups

The labial bone thickness was assessed at 4-month, and preoperative CBCT scans at the apical, middle, and coronal regions of the implant/root. Significant intra-group improvements from baseline to 4-months were observed in all thirds (apical, middle, and coronal) for both groups (*P* = 0.005^*^ for all segments). However, inter-group comparisons reveal insignificant differences (Table 5) (Fig. 4).

**Table 5:** Labial Bone Thickness analysis (mm) in the laser-stimulated and the control groups

Labial Bone Thickness (mm)			LBS+ (n=10)	LBS- (n=10)	p^1^
Apical	Baseline Preoperative Scan	Mean ±SD	0.90 ±0.33	1.14 ±0.21	0.067
		Median	0.91	1.20	
		Min – Max	0.56 – 1.45	0.86 – 1.45	
	4-monthPostoperative Scan	Mean ±SD	1.27 ±0.39	1.25 ±0.22	0.649
		Median	1.33	1.30	
		Min – Max	0.72 – 1.71	0.96 – 1.50	
	P^2^		0.005*	0.005*	
Middle	Baseline Preoperative Scan	Mean ±SD.	0.99 ±0.23	1.41 ±0.73	0.878
		Median.	1.06	1.05	
		Min – Max.	0.56 – 1.20	0.55 – 2.50	
	4-month Postoperative Scan	Mean ±SD.	1.41 ±0.41	1.75 ±0.67	0.282
		Median.	1.60	1.70	
		Min – Max.	0.67 – 1.70	0.85 – 2.58	
	P^2^		0.005*	0.005*	
Coronal	Baseline Preoperative Scan	Mean ±SD.	0.86 ±0.20	1.11 ±0.21	0.204
		Median.	1.01	1.01	
		Min – Max.	0.60 – 1.02	1.01 – 1.50	
	4-monthPostoperative Scan	Mean ±SD.	1.47 ±0.53	1.23 ±0.26	0.282
		Median.	1.50	1.10	
		Min – Max.	0.72 – 2.01	1.03 – 1.70	
	P^2^		0.005*	0.005*	

mm: millimeter; LBS+: Study group with laser application; LBS-: Control group with sham laser application; P^1^: Mann Whitney U test; P^2^: Wilcoxon Signed Rank test; *Statistically significant difference at P-value <0.05

## Discussion

Preservation of the extraction socket remodeling results in marked changes in dimensions of the alveolar ridge with an average of 0.7–1.5 mm of vertical and 4.0–4.5 mm of horizontal bone loss. Most of these dimensional alterations take place in the first 3 months following tooth extraction [6]. A plethora of modalities are proposed in order to improve the success rate of the immediate implant placement [5]. Low-Level-Laser Therapy (LLLT) has been proposed as an approach for accelerating and improving the hard and soft tissues healing process. The light irradiation has a biological and photochemical energy stimulatory interaction with conductive effects on cellular repair, wound healing, fibroblast proliferation, and collagen synthesis [6–9]. The study objective was to evaluate the effect of low-level diode laser bio-stimulation as an adjacent biostimulation for immediately placed implants in the maxillary premolar zone, with emphasis on implant stability, early and late socket healing, and radiographic appraisal.

Standardization and rigorous inclusion criteria selection parameters were set in this study. The study included only those with an expected class I socket based on both the Elian, for single-rooted teeth, or Chang et al., for maxillary premolars, classifications [14, 15]. Differences in the socket type require different surgical protocols and treatment modalities, which could act as a confounding factor during the assessment of the effect of the LLLT on wound and socket bone healing [14, 15]. All of the enrolled patients had intact post-extraction socket walls, with thick and intact gingival biotypes. Cases where the bony socket integrity was compromised during extraction were excluded. Moreover, smokers, uncontrolled diabetic patients, and patients with periapical pathology were excluded because they could complicate the surgery and affect healing, leading to implant failure.

In the study (LBS+) group, a 660-nm infrared diode laser was applied in two sessions, 7-days apart, in a non-contact mode and perpendicular beam application at buccal and palatal points. This methodology was proposed by Camolesi et al. and several previous reports, which demonstrated the beneficial effect on both the deeper and superficial tissues [17]. A gene expression analysis by Park and Kang investigated the histological observations regarding the effect of 660-nm diode laser irradiations in the reduction of inflammatory cells and accelerated bone regeneration with the proliferation of fibroblasts and osteoblasts [16].

Chen et al. (2019) report a lack of literature consensus regarding the application of laser photobiomodulation, with variation in their clinical and radiographic effects [25]. Torkzaban et al. (2017) utilized multiple sessions and a longer laser wavelength, 830–940 nm [26]. On the other hand, Lobato et al. (2020) and Kinalski et al. (2021) utilized a single, operative day, laser irradiation [27, 28]. Matys et al.(2019) demonstrated the application of multiple sessions of lower 635-nm wavelength [29]. The study utilized an overall exposure time of 100 s per session. This was based on a systematic review by Chen et al. (2019) that reported stronger bio-modulatory effects with exposure timing ranging from 30 to 120 s [25].

The LLLT Biostimulator effect on the clinical performance was documented in the study using a qualitative dichotomous manner, and a quantitative Healing Index (Hi) scale. Incidence of early postoperative complications was significantly better in the LBS+ group, where none of the patients reported any complications or signs of inflammation, in comparison to four cases in the LBS- group (*P* < 0.001). The quantitative Hi assessment reported significantly higher scores in the LBS+ group compared to the control group at all follow-up points.

Postsurgical healing is a complex procedure of multiple cellular and signaling molecules interactions. Inflammatory Signaling factors, fibroblast migration, and cellular adhesion are considered the early stages of wound healing. Other inflammatory cytokines-mediated series of events and immune cells growth factors release that occur throughout the healing process [1]. It’s proven that the adjunct utilization of low laser irradiation has an improved chemotactic effect on the motility of the epithelial cells and fibroblast migration and proliferation [6, 8]. This is correlated to the outstanding early clinical behavior regarding the LBS+ group. Furthermore, Jawad et al. outlined that laser photobiomodulation improves local application area revascularization and gingival blood flow, which could be culpable for the early improvement in the healing process [6, 8]. The results of the subjective dichotomous wound healing complications assessment could point out that there is no late difference in the 14 & 21-day incidence of wound complications, as none of the patients in the LBS- group showed any signs of inflammation or wound healing complications. However, the quantitative Hi analysis may justify the two-session laser irradiation protocol from a comprehensive gingival tissue healing and wound healing.

The pain level in our study was recorded by the visual analogue scale (VAS) scale. All patients had early mild pain, which went away entirely within 14 days. The differences were statistically significant between preoperative and after 14-days postoperative (*P* < 0.001). Hamouda et al. explained that LLLT contributes to much less discomfort experienced by the patient following surgical treatment [30]. Intra-group pain reporting difference was insignificant. The negligible laser biostimulator effect on the reported experienced pain could be attributed to the innate mild and bearable nature of the immediate implant placement procedure, as demonstrated by Fu et al. [20].

Peri-implant tissue formation occurs immediately after implant placement, and the stability of both peri-implant soft and hard tissues is considered crucial for the long-term success of implant treatment [1, 24]. Peri-implant soft and hard tissue stability and architecture after implant placement could be influenced by factors such as soft tissue quality and quantity, the type of surgical procedure, and the design of the prosthesis [22].

The final esthetics of the single crown prosthesis was quantitatively and objectively evaluated using the Pink Esthetic Score (PES) [22]. Both groups reported mean values of 10.60 ± 0.84 and 10.60 ± 0.52 in the LBS + and LBS- groups, respectively. The PES outcome is comparable to that reported by Elaskary et al. [1]. The insignificant esthetic outcome difference between both groups could be attributed to the standard provisionalization technique used in both of the investigated groups in this study. The immediately performed customized healing abutment was fashioned with respect to the biological concepts of gingival tissue attachment and emergence profile creation, which allowed proper tissue attachment [31].

Analysis of implant stability was performed in this study at the time of implant placement, primary stability, and after 4-months during the prosthetic phase, secondary stability. While the difference in the primary stability was insignificant, the LBS+ group reported significantly higher mean values of implant secondary stability. Primary stability is prominently influenced by the utilized extraction protocol, the osteotomy preparation methodology, the initial degree of bone-implant engagement, and the chosen macro-structure implant design [17]. The study opted for standardization of the majority of the primary stability affecting factors between both groups. A single-blinded operator performed the surgery on all of the enrolled patients in the study. These cumulative efforts could explain the lack of intragroup difference in the reported primary stability.

Regarding the achieved viable bone-to-implant contact and the reported secondary stability, the LBS+ group conveyed significantly higher ISQ values than the LBS- group with a 2.3-times loftier percentage of change from the primary stability (*P* < 0.001*). Gulati et al. proposed that the low infrared light irradiation has a stimulatory effect on the osteoblast precursor cell proliferation, perfusion, and an increase in their number. LLLT also has an impact on the increased local blood circulation, enriching the nutrients and oxygen inflow, which could be attributed to the histologically observed increase in bone formation and early bone healing [12, 32].

Despite the great difference in the ISQ reported values in the LBS+ group, the control group achieved loading threshold values. A similar outcome was achieved by Kim et al., where 12th week ISQ values calculated 73.3 ± 6.6 and 65.7 ± 9.7 in the laser-irradiated and the control groups, respectively [33]. Camolesi et al. assessed implant stability in a monthly manner and compared the ISQ values between laser bio-stimulated and control groups [17]. They reported the positive effect of photobiomodulation in lessening the expected early dip in implant stability, especially in the early 10–15 days, which is related to the early increase in bone activity and implant-surface remodeling [17]. Despite that, in both groups of these studies, the achieved ISQ values were lower than those achieved in this study. The study opted for a conclusive prosthesis-loading time implant stability analysis so that the bone-implant surface deposition processes are not interrupted.

To the best of the authors’ knowledge, this is the first report to demonstrate the radiographic effect of laser photobiomodulation effect on immediately placed maxillary premolar implants. Comprehensive radiographic appraisal was performed in this study using three different apparatuses. The labial bone thickness was measured for the assessment of the overall survival of the labial bone plate, the width of the alveolar process at the crestal edge was gauged as a significant predictor for the long-term prognosis of the implant, and finally mean bone density was calculated for the evaluation of the quality of the newly produced bone.

Labial bone thickness analysis reported a significant intra-group improvement from baseline to 4-months in all thirds (apical, middle, and coronal) for both groups (*p* = 0.005). However, inter-group comparisons reveal insignificant differences. A similar outcome was reported by Chu et al. [24]. The gain in the reported coronal bone labial plate thickness demonstrates the favorable preservation of bone that occurred during the extraction process, along with the favorable effect of the use of immediate implant.

Radiographic width of the alveolar process at the crestal edge reports a significantly lesser percentage of reduction in the LBS+ group when comparing the 4-months to the baseline values, indicating that less crestal bone resorption had occurred around irradiated implants. Gulati et al. assessed the impact of LLLT on peri-implant crestal bone levels at multiple time points up to one year post-loading, and reported that the LLLT group experienced significantly less crestal bone loss compared to the control group [32]. The favorable crestal bone preservation may be a good clinical prognostic indicator for the survival of the immediate implant. Khadra reports the favorable laser irradiation impact on the tissue-titanium implant attachment, which potentially contributes to the preservation of crestal bone levels [7].

The reported increase in the 4-month mean bone density showed a statistically insignificant comparable percentage of increase when comparing both groups. In an experimental study conducted by Romão et al., significant differences were observed in the percentage of newly formed bone volume and the implant stability index after applying diode laser irradiation [34]. Despite that, their study was experimental and conducted over a 6-week period. A spectroscopy analysis conducted by Lopes et al. also showed a significant difference in the concentration of calcium hydroxyapatite in the irradiated group as compared to the control group [35].

The radiographic appraisal in the study may point to the prominent effect of laser photobiomodulation on early bone-implant healing, which has a correlation to the maintenance of implant stability and the enhancement of osseointegration. According to Lopes et al., the pronounced effects of LLLT during the early stages of bone healing can be attributed to the fact that during the early stages of bone healing, the cellular component is more prominent and more prone to being affected by LLLT [35].

The inability to standardize implant diameters across all cases represents a limitation that may have influenced radiographic outcomes. To mitigate bias, however, most other confounding factors were standardized, and the study was localized to the maxillary premolar region. Additional limitations include the high standard deviation observed in the percentage of ISQ change. Furthermore, the clinical nature of the study lacked a histological evaluation for the gingival healing on a cellular level, specifically at the irradiation target areas.

Within the confines of the study, the utilization of low-level laser biostimulation has improved early clinical outcomes with an improved wound healing performance, especially with the contemporary drive for immediate implant provisionalization and customization. The long-term outcomes in the study verify the sufficiency of the double laser-irradiation protocol. As an adjunct to immediate implant placement, LLLT may reduce early postsurgical inflammation and enhance soft tissue healing. While early-phase results were positive, overall success rate, osteointegration, secondary implant stability, and radiographic performance were comparable to the control group with no extra benefits, suggesting that the biostimulatory effects of the laser are primarily limited to the early healing phase.

## Data Availability

All of the analyzed data in this study are available upon reasonable request.

## Abbreviations

LLLT: Low-Level-Laser Therapy
LBS+: Study Group (Immediate implant placement with laser application)
CBW: Crestal Bone With
RFA.: Radiofrequency Analysis.
SPSS.: Statistical Package for Social Sciences.
VAS: Visual Analogue Scale
Hi.: Landry Healing Index
CBCT: Cone Beam Computed Tomography
LBS-: Control Group (Immediate implant placement with sham laser application).
GSV: Grey Scale Value
ISQ: Implant Stability Quotient
SNOSE: Sequentially numbered, opaque, sealed envelopes
PES: Pink Esthetic Score
